# Years of life lost estimates cannot always be taken at face value: Response to “COVID-19 – exploring the implications of long-term condition type and extent of multimorbidity on years of life lost: a modelling study”

**DOI:** 10.12688/wellcomeopenres.16015.1

**Published:** 2020-06-11

**Authors:** Marius Rubo, Peter Czuppon

**Affiliations:** 1 Department of Psychology, University of Fribourg, Fribourg, 1700, Switzerland; 2 Institute of Ecology and Environmental Sciences, Sorbonne Université, Paris, 75252, France; 3 Centre Interdisciplinaire de Recherche en Biologie, Collège de France, Paris, 75005, France

**Keywords:** COVID-19, Mortality Burden, Years of Life Lost, Data Selection Bias

## Abstract

In their recent analysis, Hanlon *et al*. set out to estimate the years of life lost (YLL) in people who have died with COVID-19 by following and expanding on the WHO standard approach. We welcome this research as an attempt to draw a more accurate picture of the mortality burden of this disease which has been involved in the deaths of more than 300,000 people worldwide as of May 2020. However, we argue that obtained YLL estimates (13 years for men and 11 years for women) are interpreted in a misleading way. Even with the presented efforts to control for the role of multimorbidity in COVID-19 deaths, these estimates cannot be interpreted to imply “how long someone who died from COVID-19 might otherwise have been expected to live”. By example we analyze the underlying problem of data selection bias which, in the context of COVID-19, renders such an interpretation of YLL estimates impossible, and outline potential approaches to control for the problem.

## Introduction: The debate around COVID-19’s mortality burden

Hanlon *et al*. (2020) motivate their modelling study with the observation that raw death counts can exaggerate the mortality burden of COVID-19 (by weighing equally the death of elderly people, whose life expectancy may only be several years, with those of younger people who might otherwise expect to continue to live for decades), while, on the other hand, some statements in the public media have likely underestimated the mortality burden (by simply emphasizing the proximity of age of death in people dying with COVID-19 to that of people dying in the general population, neglecting the fact that even people who have surpassed the average age of death can usually expect to continue to live for years). The authors then propose to calculate years of life lost (YLL) using WHO life tables to more accurately describe the burden of mortality. In introducing this approach, the authors start with the correct assumption that “YLL can be expressed per-capita as the average number of years an individual would have been expected to live had they not died of a given cause”. While this idea may seem immediately intuitive, “it is neither simple to compute nor to comprehend” ( Gardner & Sanborn, 1990, p. 328). Reckoning that, outside of idealized models, the cause of death is only ever given within degrees of uncertainty, we will argue here that YLL estimates can be taken at face value only if a) there exists a causal model which allows to precisely describe the interaction of factors which led to a person’s death (and not only the fact that a factor of interest is unequivocally involved) or b) the age of death in the population of interest is much smaller than the age of death in the general population. Neither of these enabling conditions are given in the context of COVID-19.

## Years of life lost estimates elucidate in some situations, mislead in others

The goal of this comment is to develop a qualitative, conceptual understanding of why the interpretation of YLL estimates rests on specific preconditions and why they are not met in the case of COVID-19. For this purpose, we will first introduce two hypothetical examples, one where the interpretation of YLL estimates is clearly correct and one where it is clearly misleading.

An example where the interpretation of YLL estimates would be correct could be that of a man who dies from a brain tumor at the age of 40. When we know that, in the specific country, men who have lived to the age of 40 will, on average, continue to live until 85, it seems fair to infer that the brain tumor has cost this person about 45 years of his life. More detailed analyses may additionally incorporate other variables from the deceased. If we know that he exercised regularly, did not smoke but suffered from some long-term conditions (LTCs) such as Diabetes, we may specifically look for a reference group of other men from the same country matched on these variables and see how long they, on average, continued to live after they had reached the age of 40. This more accurate comparison will give us an even better estimate of the YLL in the specific case, but no matter how many variables we include when defining a reference group, it will typically not differ strongly from the original estimate as there are no common preconditions which very drastically reduce life expectancy of a 40-year-old to, say, as little as 10 years.

For an example where the calculation of YLL would be clearly misleading, consider a hypothetical town where a previously little-known exotic fruit named jackfruit gains popularity. If a scientist were to investigate the hypothesis that eating jackfruits is bad for people’s health and results in premature deaths, she could collect data from all people who died in this town and were known to regularly eat this fruit, and compute YLL for each of them. For instance, when a person died aged 82, she reads from a life table that other people who lived to be 82 would then, on average, continue to live for another 8 years, and notes this value as YLL for the specific person. She also refines the model by restricting the reference group along a variety of health-related variables, but still obtains positive YLL values (she *necessarily* does, because she still compares each person with other people who lived *at least* as long ( Bonneux, 2002; Marshall, 2010)) and considers the hypothesis proven.

The interpretation of YLL estimates should be reasonable in the case of the man who died of a brain cancer since we can assume that his death can be mono-causally attributed to a specific cause. There should not be much uncertainty around this attribution, since 1) there is a clear and observable causal model of how brain tumors can end the life of a person regardless of other health parameters and 2) other natural and possibly unobserved or unknown causes of death at the age of 40 are so rare that they may reasonably be ruled out.

By contrast, the scientist investigating the effect of jackfruits starts out with a (as we would argue) wrong assumption (that eating jackfruits leads to premature deaths), but this assumption is never corrected in the process of calculating YLL values. The lesson here is that you have to get the cause of death right *prior* to performing a YLL calculation. Note that defining the reference group ever more precisely will reduce, but never eliminate the problem that cases are selected based on the mere fact that they have died rather than on the fact that they have died of a specific given cause. Even if people who died after regularly eating jackfruits are compared against others who are matched on an abundance of variables (say, a hundred variables known to predict longevity), they would still be the youngest to die in their reference group. It is because of this selection bias that the individuals in a YLL analysis, where the assumed cause of death is incorrect or not entirely correct, can never be representative of their reference group.

## Any uncertainty around the cause of death inflates years of life lost estimates

We have seen that YLL estimates can provide a reasonable and intuitive measure for how many years a person has lost due to a specific cause of death, but that these estimates can also be wildly misleading when applied regardless of their constraints. Next, we intend to outline how well the situation surrounding COVID-19 may fit the constraints needed to obtain meaningfully interpretable YLL estimates.

While the cause of death assumed for the YLL analysis in the jackfruit example was not associated at all with the true cause of death, there is wide agreement that COVID-19 does in fact play an important role in the deaths of people who die while suffering from COVID-19. Crucially, however, this observation is not identical with the assumption that COVID-19 was *the* cause of death. Hanlon *et al*. (2020) themselves acknowledged that “people dying from COVID-19 are predominantly older and have pre-existing LTCs” (citing, among others, Zhou *et al*. (2020)), but did not acknowledge that this observation must be integrated not only in how reference groups in a YLL analysis are defined, but also into how the cause of death itself is defined. In the presence of such moderating or additive effects with other variables, the cause of death must be viewed as a combination of COVID-19 with other preconditions. Crucially, as the assumed cause of death in this YLL analysis (people were mono-causally killed by COVID-19) *partly* misses the true cause of death (COVID-19, together with other known and unknown preconditions, led to people’s death), people in this analysis are also selected *partly* on the mere grounds of having died rather than on the grounds of having died from COVID-19. As we have seen, selecting cases on the mere grounds of having died rather than of having died from a given cause has one specific effect: it unidirectionally inflates resulting YLL values. This observation may seem counter-intuitive considering that in many applications of statistics, adding uncertainty to an underlying variable will increase the uncertainty around resulting estimates, but will often not bias estimates in a specific direction. The situation is different here. Knowing that we cannot precisely predict the death of people suffering from COVID-19 even when employing all available data on sex, age and LTCs (a remark which would seem trivial in most other contexts), we must infer that there exists residual and/or unmeasured confounding ( Fewell *et al*., 2007) which has additionally contributed to the fact that these individuals were the first to die within their reference group (in other words, the effect of these unknown confounds must have had a life-shortening rather than a life-extending effect on these individuals, relative to their reference group).

Since it is quite common that the cause of death of a person is not *perfectly* known, one may ask why YLLs are used in epidemiology at all. Firstly, the problem of a selection bias resulting from uncertain causes of death is existent, but much less salient when the average age of death in a particular group is clearly smaller compared to the age of death in the general population. If a disease frequently kills 40-year-old individuals, the underlying mechanism that leads to the death may of course also incorporate an interaction of the disease with other (partly unknown) preconditions. However, since a combination of any such preconditions with any other diseases (be they known or unknown) only very rarely leads to a death among this age group, the cause of death can be more strongly attributed to the disease even in the absence of a precise causal model. The rationale behind such causal attributions have been described in general by Cheng & Novick (1992) and Pearl (2009) and were investigated in the domain of epidemiology by Suzuki *et al*. (2012). We assume that Gardner & Sanborn (1990) – whom Hanlon *et al*. (2020) cite – do not themselves elaborate on this topic since they noted that YLL “has been promoted to emphasize specific causes of death affecting younger age groups”. Secondly, several publications have employed YLL estimates not with the goal of interpreting them directly, but rather to compare them between different diseases (e.g. Murray *et al*., 2012). This application (which we would deem valid given the amount of uncertainty around each disease’s role in people’s death is comparable) is present in the Hanlon *et al*. (2020) study, too. Here, however, the authors furthermore interpret YLL estimates directly, which is the object of our criticism.

Considering that the uncertainty surrounding the exact cause of death in patients who died with COVID-19 is well-documented (simply by our inability to precisely predict who will die while suffering from COVID-19 and who will not), and further considering that any deviation of the assumed cause of death from the true cause of death leads to a selection bias in the context of a YLL analysis (especially when people of interest died at an older age), we were perplexed to read that the authors treated those dying from COVID-19 as representative of their reference group. While the authors acknowledged that they “did not have markers of underlying disease severity among those who died” and that they “had no data for rarer severe LTCs”, they nonetheless asserted that “this effect is unlikely to be substantial enough to reduce YLL to the orders of magnitude suggested by some commentators.” The authors furthermore stated during the open review process that “in order to make the assertion that those dying from COVID19 are atypical of their fellows who are similar in terms of age, sex and comorbidity we would argue that empirical evidence to support that claim is needed”. As we have seen above, those who died with COVID-19 are *a priori* atypical of their fellows to the extent that their cause of death is uncertain. The authors’ efforts in defining reference groups as narrowly as possible by including LTCs may reduce, but can never eliminate this fundamental problem.

## Other ways to deal with this irresolvable problem

In our view, a more meaningful approach to investigating the burden of mortality due to COVID-19 is to compare monthly mortality curves normalized over the age classes from the last decade to the excess in corresponding mortality curves as obtained since the beginning of the pandemic. Computing the average age of death for both curves will give a reasonable estimate in case we are sure that the difference is *exclusively* attributable to the ongoing pandemic (but not necessarily to COVID-19 in a mono-causal manner). A similar approach has been proposed to estimate the excess in overall mortality due to COVID-19 ( Leon *et al*., 2020). Admittedly though, data of this type are rarely accessible.

If one wishes to continue using YLL estimates in the context of COVID-19 and interpret them directly (which we think appears tempting when the boundary conditions for a direct interpretability are met), we suggest correcting for the uncertainty surrounding the exact cause of death. Marshall (2010) estimated YLL values for the general population (when no cause of death is specifically assumed – an assumption which is functionally equivalent to assuming a random cause of death) to be around 9–10 years and proposed for YLL estimates concerning specified causes of death that “if years of life lost per death is calculated to be about 9–10 years, it is not out of the ordinary and means that the age at death is congruent to the MLTW age structure” (p. 407). Applying this approach to the life table of the WHO for Italy from 2016 ^1^ results in 9.5 and 8 YLL for men and women, respectively. Subtracting these baseline YLL values from the uncorrected cause-specific YLL estimates found by Hanlon *et al*. (2020) we obtain 4.5 and 4 YLL for men and women, respectively (see GitHub and *Extended data* for scripts and data source ( Czuppon & Rubo, 2020)). However, we would argue that subtracting such a YLL baseline from obtained YLL values would not yield fair estimates in the case of COVID-19 either since it seems that with this disease, especially elderly people are seriously affected. Consider another hypothetical example here: if a serial killer were to specifically murder elderly people (say, above the mean age of death) and one subtracted such a YLL baseline from the victims’ YLL values, one would obtain negative YLL estimates (which would, if interpreted at face value, indicate that being murdered by that serial killer prolongs one’s life). We would argue that in this case, no correction to the obtained YLL values is needed as there is virtually no uncertainty around the cause of death (the murder mono-causally killed the victims, signifying that if it were not for the murder, the victims could be assumed to be representative for their reference groups). On the other hand, we would suggest subtracting 100% of the baseline from YLL values obtained in the jackfruit example as we cannot reasonably attribute any causal effect here. More generally, we would suggest correcting for a possible data collection bias by subtracting a fraction of such a YLL baseline depending on the amount of uncertainty surrounding the cause of death (see also the literature about exposed YLL estimations, e.g. Hammitt *et al*. (2020)). Note that using such an approach, the correctness of YLL estimates would now hinge on the correctness of uncertainty estimates regarding the cause of death – a precondition which we consider irresolvable. However, since such an estimation of uncertainty could both under- and overestimate the true value, this approach would at least eliminate a unidirectional bias which can only ever inflate YLL estimates.

It has been shown in a variety of cases that standard measures in epidemiology can be, and are, frequently used in misleading ways, even by medical experts ( Gigerenzer *et al*., 2007). We argue that this is also true for the study by Hanlon *et al*. (2020). By not properly accounting for the uncertainty around the precise cause of death, the study is unprotected against a bias in data selection, leading to YLL estimates which must be considered inflated if one interprets them directly, as the authors do. The authors themselves noted that their study was “conducted rapidly and under pressure of time”. In our personal view, these circumstances may explain rushed conclusions, and we express respect to the huge effort that was taken under these circumstances. Nonetheless, we suggest to revise the interpretation of the data at hand and to prevent misleading information from further spreading into the public.

## Data availability

### Underlying data

All data underlying the results are available as part of the article and no additional source data are required.

### Extended data

**Python scripts alongside all necessary data tables available at:** https://github.com/pczuppon/YLL_computation.

**Archived scripts at time of publication:** http://doi.org/10.5281/zenodo.3874662 ( Czuppon & Rubo, 2020).

**License:** Creative Commons Attribution 4.0 International.

## Footnotes

^1^ https://apps.who.int/gho/data/view.main.60800?lang=en (accessed 25/05/2020)
