# Years of life lost estimates cannot always be taken at face value: Response to “COVID-19 – exploring the implications of long-term condition type and extent of multimorbidity on years of life lost: a modelling study”

**DOI:** 10.12688/wellcomeopenres.16015.2

**Published:** 2022-01-13

**Authors:** Marius Rubo, Peter Czuppon

**Affiliations:** 1 Department of Psychology, University of Fribourg, Fribourg, 1700, Switzerland; 2 Institute of Ecology and Environmental Sciences, Sorbonne Université, Paris, 75252, France; 3 Centre Interdisciplinaire de Recherche en Biologie, Collège de France, Paris, 75005, France; 4 Institute for Evolution & Biodiversity, University Münster, 48149 Münster, Germany

**Keywords:** COVID-19, Mortality Burden, Years of Life Lost, Data Selection Bias

## Abstract

In their recent analysis, Hanlon *et al*. estimated the years of life lost (YLL) in people who have died with COVID-19 by following and expanding on the WHO standard approach. We welcome this research as an attempt to draw a more accurate picture of the mortality burden of this disease which has been involved in the deaths of more than 300,000 people worldwide as of May 2020. However, we argue that obtained YLL estimates (13 years for men and 11 years for women) are interpreted in a misleading way. Even with the presented efforts to control for the role of multimorbidity in COVID-19 deaths, these estimates cannot be interpreted to imply “how long someone who died from COVID-19 might otherwise have been expected to live”. By example we analyze the underlying problem which renders such an interpretation of YLL estimates impossible, and outline potential approaches to control for the problem.

## Introduction: The debate around COVID-19’s mortality burden

Hanlon *et al*. (2020) motivate their modelling study with the observation that raw death counts can exaggerate the mortality burden of COVID-19 (by weighing equally the death of elderly people, whose life expectancy may only be several years, with those of younger people who might otherwise expect to continue to live for decades), while, on the other hand, some statements in the public media have likely underestimated the mortality burden (by simply emphasizing the proximity of age of death in people dying with COVID-19 to that of people dying in the general population, neglecting the fact that even people who have surpassed the average age of death can usually expect to continue to live for years). The authors then propose to calculate years of life lost (YLL) using WHO life tables as a means to express the “average number of years an individual would have been expected to live had they not died of a given cause”.

## Years of life lost estimates elucidate in some situations, mislead in others

The attribution of YLL estimates to a person’s cause of death is complicated by the fact that YLL estimates are always positive and were found to amount to 9–10 years in the general population ( Marshall, 2010). This observation led Marshall (2010) to conclude that “if years of life lost per death is calculated to be about 9–10 years, it is not out of the ordinary and means that the age at death is congruent to the MLTW [Model Life Table West] age structure”. For this reason, YLL estimates are often merely compared between diseases (e.g. Murray *et al.*, 2012) rather than interpreted directly (i.e., to imply how long someone who died might have been expected to live). Since Hanlon *et al.* (2020) aimed for an interpretation of YLL estimates at face value, we illustrate here using hypothetical examples how YLL estimates can be interpreted.

As an example where YLL estimates can be interpreted directly, consider a man who dies from a brain tumor at the age of 40. When we know that, in the specific country, men who have lived to the age of 40 will, on average, continue to live until 85, it seems fair to infer that the brain tumor has cost this person about 45 years of his life. More detailed analyses may additionally incorporate other variables from the deceased. If we know that he suffered from a long-term condition (LTC) such as Diabetes, we may specifically look for a reference group of other men from the same country matched on this variable and see how long they, on average, continued to live after they had reached the age of 40. This more accurate estimate will typically not differ strongly from the original estimate as there are no common preconditions which very drastically reduce life expectancy of a 40-year-old to, say, as little as 10 years.

For an example where the calculation of YLL would be clearly misleading, consider a hypothetical town where a previously little-known exotic fruit named jackfruit gains popularity. If a scientist were to investigate the hypothesis that eating jackfruits is bad for people’s health and results in premature deaths, she could collect data from all people who died in this town and were known to regularly eat this fruit, and compute YLL for each of them. For instance, when a person died aged 82, she reads from a life table that other people who lived to be 82 would then, on average, continue to live for another 8 years, and notes this value as YLL for the specific person. She obtains an average YLL estimate of 10 years for people who ate jackfruits and concludes that the fruit shortens people’s lives.

The interpretation of YLL estimates should be reasonable in the case of the man who died of a brain cancer since we can assume that his death can be attributed to a specific cause. There should not be much uncertainty around this attribution, since 1) there is a clear and observable causal model of how brain tumors can end the life of a person regardless of other health parameters and 2) other natural and possibly unobserved or unknown causes of death at the age of 40 are so rare that they may reasonably be ruled out. By contrast, the scientist investigating the effect of jackfruits starts out with a (as we would argue) wrong assumption (that eating jackfruits leads to premature deaths), but this assumption is never corrected in the process of calculating YLL values. Note that defining the reference group ever more precisely will reduce, but never eliminate the problem. Even if people are matched on multiple variables (say, a hundred variables known to predict longevity), they would still be the youngest to die in their reference group.

While the cause of death assumed in the jackfruit example was not associated at all with the true cause of death (and serves here only to exemplify that even a wrongly assumed cause of death may be associated with substantial YLLs), there is wide agreement that COVID-19 does in fact play an important role in the deaths of people who die with COVID-19. At the same time, COVID-19 does not lead to a person’s death in a virtually monocausal manner as it can be observed with brain tumors. Instead, as indicated in the older age and presence of pre-existing LTCs in people dying with COVID-19, the disease interacts with poor prior health in causing an individual’s death. A somewhat similar relationship is seen in the often lethal effect of *Pneumocystis jirovecii* specifically in people with AIDS ( Tellez *et al.*, 2008). To account for the role of interacting factors in causing the death of COVID-19 patients, Hanlon *et al.* (2020) therefore adjust their analysis for the number of LTCs, consequently observing a reduction in YLLs. Note that if reference groups were defined more narrowly (perhaps incorporating the type or severity of LTCs), YLL estimates can be expected to further decrease, but not increase: when people are grouped more precisely along risk factors, the variance in their remaining lifetime shrinks (as its predictability increases), resulting in a smaller average difference in remaining lifetime for each dying person and other people who got to live at least as long.

## Interpreting YLL estimates

To avoid partially misattributing YLL estimates to an individual factor, when in fact a combination of factors led up to people’s deaths, one could compare YLL estimates in a group of interest (people who died with COVID-19) with those in the general population. Applying the approach of Marshall (2010) to the life table of the WHO for Italy from 2016 ^1^ results in 9.5 and 8 YLL for men and women, respectively. Subtracting these baseline YLL values from the uncorrected cause-specific YLL estimates found by Hanlon *et al*. (2020) we obtain 4.5 and 4 YLL for men and women, respectively (see GitHub and *Extended data* for scripts and data source ( Czuppon & Rubo, 2020)). However, we would argue that subtracting such a YLL “baseline” from obtained YLL values could also be misleading in the case of COVID-19 where especially elderly people are seriously affected. Consider another hypothetical example here: if a serial killer were to specifically murder elderly people (say, above the mean age of death) and one subtracted such a YLL baseline from the victims’ YLL values, one would obtain negative YLL estimates (which would, if interpreted at face value, indicate that being murdered by that serial killer prolongs one’s life). We would argue that in this case, no correction to the obtained YLL values is needed as there is virtually no uncertainty around the cause of death (the murder mono-causally killed the victims, signifying that if it were not for the murder, the victims could be assumed to be representative for their reference groups). On the other hand, we would suggest subtracting 100% of the baseline from YLL values obtained in the jackfruit example as we would not attribute any causal effect here.

More generally, when YLL estimates are to be interpreted directly, we suggest to subtract a fraction of such a YLL baseline depending on the amount of uncertainty surrounding the cause of death (see also the literature about exposed YLL estimations, e.g. Hammitt *et al*. (2020)). The rationale behind causal attributions have been described in general by Cheng & Novick (1992) and Pearl (2009) and were investigated in the domain of epidemiology by Suzuki *et al.* (2012). Problematically, however, the data available to Hanlon *et al.* (2020) do not allow for a thorough analysis of causal links between COVID-19, other health factors and people’s deaths. We therefore suggest to use obtained YLL estimates only for the purpose of comparing them with those in other studies and to avoid a direct interpretation, which would be misleading.

An alternative approach to investigate the burden of mortality due to COVID-19 is to compare monthly mortality curves normalized over the age classes from years prior to the pandemic to the excess in corresponding mortality curves as obtained since the beginning of the pandemic. Computing the average age at death for both curves can give a reasonable estimate in case we are sure that the difference is exclusively attributable to the ongoing pandemic (but not necessarily to COVID-19 in a direct manner). A similar approach has been proposed to estimate the excess in overall mortality due to COVID-19 ( Leon *et al.*, 2020).

## Data availability

### Underlying data

All data underlying the results are available as part of the article and no additional source data are required.

### Extended data

**Python scripts alongside all necessary data tables available at:** https://github.com/pczuppon/YLL_computation.

**Archived scripts at time of publication:** http://doi.org/10.5281/zenodo.3874662 ( Czuppon & Rubo, 2020).

**License:** Creative Commons Attribution 4.0 International.

## Footnotes

^1^ https://apps.who.int/gho/data/view.main.60800?lang=en (accessed 25/05/2020)
