# Supplementary material for: Years of life lost estimates cannot always be taken at face value: Response to “COVID-19 – exploring the implications of long-term condition type and extent of multimorbidity on years of life lost: a modelling study”
Source: Wellcome Open Res. 2022 Jan 13;5:137. Originally published 2020 Jun 11. [Version 2] doi: 10.12688/wellcomeopenres.16015.2 (PMC8874032; doi:10.12688/wellcomeopenres.16015.2)
Supplement: Peer review history

# Peer review: Reviewer response for version 2

**DOI:** 10.21956/wellcomeopenres.19450.r48029

**Published:** 2022-02-24

**Authors:** David A. Leon

**Affiliations:** 1 Faculty of Epidemiology and Population Health, London School of Hygiene and Tropical Medicine, London, UK

The authors have revised their critique of Hanlon *et al.,* in response to reviewers' comments. Their piece is now shorter and somewhat clearer. The final paragraph of the revised critique is helpful as it more explicitly lays out than before an alternative to using YLL to estimate the mortality burden of COVID using instead excess some form of excess death.

Are arguments sufficiently supported by evidence from the published literature or by new data and results?

Partly

Is the conclusion balanced and justified on the basis of the presented arguments?

Partly

Is the rationale for commenting on the previous publication clearly described?

Partly

Are any opinions stated well-argued, clear and cogent?

Partly

Reviewer Expertise:

Epidemiology, excess deaths, demographic trends

I confirm that I have read this submission and believe that I have an appropriate level of expertise to confirm that it is of an acceptable scientific standard.

# Peer review: Reviewer response for version 2

**DOI:** 10.21956/wellcomeopenres.19450.r48030

**Published:** 2022-02-14

**Authors:** Yijun Lou

**Affiliations:** 1 Department of Applied Mathematics, Hong Kong Polytechnic University, Hong Kong, China

No further comments.

Are arguments sufficiently supported by evidence from the published literature or by new data and results?

Yes

Is the conclusion balanced and justified on the basis of the presented arguments?

Partly

Is the rationale for commenting on the previous publication clearly described?

Yes

Are any opinions stated well-argued, clear and cogent?

Yes

Reviewer Expertise:

Theoretical epidemiology and mathematical biology.

I confirm that I have read this submission and believe that I have an appropriate level of expertise to confirm that it is of an acceptable scientific standard.

# Peer review: Reviewer response for version 1

**DOI:** 10.21956/wellcomeopenres.17565.r44032

**Published:** 2021-06-11

**Authors:** Yijun Lou

**Affiliations:** 1 Department of Applied Mathematics, Hong Kong Polytechnic University, Hong Kong, China

Due to the complexity and multi-causality nature involved, it is challenging to measure YLL for a given disease in a heterogeneous population. The authors highlighted some wonderful constraints in measuring/interpreting this index.

 Two examples in the section "Years of life lost estimates elucidate in some situations, mislead in others" would not be appropriate in the context of COVID-19. The first example is related to a specific individual (or a homogeneous population), while YLL is an index for the population of individuals with different characteristics and different age/death/disease distributions were used in the study by Hanlon *et al.*. The second jackfruits example is not applicable to the case for COVID-19 (which was also mentioned in page 4.)

 The authors emphasied that "The interpretation of YLL estimates should be reasonable in the case of the man who died of a brain cancer since we can assume that his death can be mono-causally attributed to a specific cause." Although YLL estimates in Hanlon *et al*. measure the direct impact of COVID-19 deaths rather than the indirect impact of COVID-19-related outcomes., the terminology YLL itself would make sense in the multi-causality case, through direct and indirect effects (as discussed in Hanlon et al.).

 In fact, the age distribution of COVID-19 deaths of COVID-19 and age model were employed in Hanlon *et al.* The hypothetical example of a serial killer may not give rise to a negative YLL value, as claimed in Page 5.

Are arguments sufficiently supported by evidence from the published literature or by new data and results?

Yes

Is the conclusion balanced and justified on the basis of the presented arguments?

Partly

Is the rationale for commenting on the previous publication clearly described?

Yes

Are any opinions stated well-argued, clear and cogent?

Yes

Reviewer Expertise:

Theoretical epidemiology and mathematical biology.

I confirm that I have read this submission and believe that I have an appropriate level of expertise to confirm that it is of an acceptable scientific standard, however I have significant reservations, as outlined above.

# Peer review: Reviewer response for version 1

**DOI:** 10.21956/wellcomeopenres.17565.r42434

**Published:** 2021-02-19

**Authors:** Mohsen Mirzaei

**Affiliations:** 1 Yazd Cardiovascular Research Center, Health monitoring research center, School of Medicine, Shahid Sadoughi University of Medical Sciences, Yazd, Iran

There are limitations mentioned by the authors for the index. This type of critique of the YLL index is correct. But it should be noted that this is not a new problem. The purpose of this index is to compare different diseases and societies. Therefore, with the same calculation method, comparison will not be a problem.

 So the jackfruits example is not appropriate. For this critique, it would be better to cite an example - a disease that is more deadly in the elderly.

 Another point mentioned in the text but not explained is that the elderly have underlying disease. This can cause comorbidity. Calculating the YLL according to the underlying disease can help to understand the authors' critique, because the estimates in YLL are based on the lack of underlying disease and this can distort the result. Overall; the indexing of this manuscript could draw the attention of others to future YLL corona calculations, and recommend that the authors write suggestions in manuscript editing so that future researchers can take the authors' advice more carefully in calculating the "Years of Life Lost" for diseases such as COVID-19. Suggest calculating a method that helps solve the problem in the calculation, not just express the problem.

Are arguments sufficiently supported by evidence from the published literature or by new data and results?

Partly

Is the conclusion balanced and justified on the basis of the presented arguments?

Yes

Is the rationale for commenting on the previous publication clearly described?

Yes

Are any opinions stated well-argued, clear and cogent?

Partly

Reviewer Expertise:

Public Health, Burden of disease, Non-Communicable disease epidemiology.

I confirm that I have read this submission and believe that I have an appropriate level of expertise to confirm that it is of an acceptable scientific standard, however I have significant reservations, as outlined above.

# Peer review: Reviewer response for version 1

**DOI:** 10.21956/wellcomeopenres.17565.r39187

**Published:** 2020-07-17

**Authors:** David A. Leon

**Affiliations:** 1 Faculty of Epidemiology and Population Health, London School of Hygiene and Tropical Medicine, London, UK

This commentary raises several questions about the use and interpretation of Years of Life Lost (YLL) that go beyond the specific paper by Hanlon *et al.* that the authors have focussed upon. The key issue is one that has already been discussed in several publications by RJ Marshall one of which is cited by Rubo & Czuppon. A more accessible paper by Marshall that makes the key relevant point was published in 2004. ^1^ Marshall points out the counter-intuitive result that YLL is substantial and positive even when calculated for the chosen reference or standard population used such as the Standard WHO lifetables used by Hanlon *et al. *in one part of their analysis. To calculate YLL in this situation you simply multiply the number of deaths from a cause of interest (or indeed all causes together) occurring in a defined age group by the conditional expectation of life at that age. This of necessity yields a number that is much higher than zero. Marshall does this for the Model Life Table West (MLTW) and shows that there is a crude YLL per death of around 9 years. For logical reasons, it cannot be zero. Importantly this means that the figure of 9 years cannot, therefore, be interpreted as showing that a population with a mortality structure of MLTW has an average age at death (per death) 9 years higher than itself! This is an unsatisfactory but inescapable property of YLLs.

 It is this paradox and limitation of YLL that must lead Rubo & Czuppon to take issue with the few places in Hanlon where they refer to a metric of “how long someone who died from COVID-19 might otherwise have been expected to live”. Clearly YLL does not provide this metric when interpreted in absolute terms. However, comparing YLLs by age and according to number of long-term conditions as Hanlon *et al. *do is meaningful – showing that YLLs decline with the extent of comorbidity and age.

 Rubo & Czuppon could have made this point in a simpler and more direct fashion. It is worth making given the original motivation of Hanlon *et al. *which was to counter claims that most people who die of COVID-19 in countries such as the UK are at an age and level of frailty that means their deaths would have been brought forward by only a small amount.

 The commentary by Rubo & Czuppon however is difficult to follow. They return repeatedly to the notion of “uncertainty around the precise cause of death”. While this cannot be disputed, I could not follow why this was an issue with respect to YLLs or their interpretation. This would need substantial clarification if it was to be retained in the commentary.

 Overall the commentary would benefit from some light touch language editing and careful proofreading (there are a number of typos). It could also be substantially shortened.

Are arguments sufficiently supported by evidence from the published literature or by new data and results?

Partly

Is the conclusion balanced and justified on the basis of the presented arguments?

Partly

Is the rationale for commenting on the previous publication clearly described?

Partly

Are any opinions stated well-argued, clear and cogent?

Partly

Reviewer Expertise:

Epidemiology, excess deaths, demographic trends

I confirm that I have read this submission and believe that I have an appropriate level of expertise to state that I do not consider it to be of an acceptable scientific standard, for reasons outlined above.

## References

[1] : Standard expected years of life lost as a measure of mortality: norms and reference to New Zealand data. *Aust N Z J Public Health* .2004;28(5) : 10.1111/j.1467-842x.2004.tb00027.x 452-7 10.1111/j.1467-842x.2004.tb00027.x 15707187

# Response

**Published:** 2020-08-05

**Authors:** Marius Rubo

**Affiliations:** University of Fribourg, Switzerland

We thank the reviewer for reading and reviewing our work.  

We are pleased to read that the reviewer agrees with us that the YLL estimates obtained in the study by Hanlon et al. cannot be interpreted to imply “how long someone who died from COVID-19 might otherwise have been expected to live”. The presence of this direct interpretation in the work by Hanlon et al. is the object of our criticism.

As the reviewer seems to agree with our main argumentation and conclusion, we are confused as to why he rated our work as not being “of an acceptable scientific standard”. We would like to note that we do not consider our manuscript as a scientific research article but as a comment (or response) to the article published by Hanlon et al. (2020). As such, we do not emphasize adding new scientific insight into the methodology of YLL estimates, but focus on describing how caveats in the interpretation of YLLs refer to the study by Hanlon et al. which has been prominently cited in the public media.  

Regarding our explanations of problems with YLL estimates, the most tangible objection to our commentary seems to be that the reviewer “could not follow why [the uncertainty around the precise cause of death] was an issue with respect to YLLs or their interpretation”. In our commentary we argue that YLL estimates can be interpreted directly (or, in the reviewer’s words, “in absolute terms”) when the cause of death is fully known. In this sense, our objection to the direct interpretation of YLLs does not merely rest on an intrinsic property of YLLs (that they can never be zero), but rather on the missing causal modelling of factors that led to people’s deaths and which would allow for an interpretation in absolute terms.

The reviewer mentions that the misleading direct interpretation of YLL estimates is present in “few places in Hanlon”. In fact, we only counted one instance where this direct interpretation is explicitly present. However, note that this misleading interpretation is already being cited in several academic works (e.g. Pearce, Lawlor & Brickley, 2020), and, following Hanlon et al.’s press release (which also contains the direct interpretation) has become the dominant theme in many of the more than 100 news reports citing the study. This is why we suggested to correct this (and only this) point in the manuscript by Hanlon et al. (2020) and to transparently discuss the interpretability of the results.

Pearce, N., Lawlor, D. A., & Brickley, E. B. (2020). Comparisons between countries are essential for the control of COVID-19. *International Journal of Epidemiology.*
